# Using individual participant data to improve network meta-analysis projects

**DOI:** 10.1136/bmjebm-2022-111931

**Published:** 2022-08-10

**Authors:** Richard D Riley, Sofia Dias, Sarah Donegan, Jayne F Tierney, Lesley A Stewart, Orestis Efthimiou, David M Phillippo

**Affiliations:** 1 School of Medicine, Keele University, Keele, UK; 2 Centre for Reviews and Dissemination, University of York, York, UK; 3 Department of Health Data Science, University of Liverpool, Liverpool, UK; 4 MRC Clinical Trials Unit at UCL, UCL, London, UK; 5 Institute of Social and Preventive Medicine (ISPMU), University of Bern, Bern, Switzerland; 6 Population Health Sciences, Bristol Medical School, University of Bristol, Bristol, UK

## Abstract

A network meta-analysis combines the evidence from existing randomised trials about the comparative efficacy of multiple treatments. It allows direct and indirect evidence about each comparison to be included in the same analysis, and provides a coherent framework to compare and rank treatments. A traditional network meta-analysis uses aggregate data (eg, treatment effect estimates and standard errors) obtained from publications or trial investigators. An alternative approach is to obtain, check, harmonise and meta-analyse the individual participant data (IPD) from each trial. In this article, we describe potential advantages of IPD for network meta-analysis projects, emphasising five key benefits: (1) improving the quality and scope of information available for inclusion in the meta-analysis, (2) examining and plotting distributions of covariates across trials (eg, for potential effect modifiers), (3) standardising and improving the analysis of each trial, (4) adjusting for prognostic factors to allow a network meta-analysis of conditional treatment effects and (5) including treatment–covariate interactions (effect modifiers) to allow relative treatment effects to vary by participant-level covariate values (eg, age, baseline depression score). A running theme of all these benefits is that they help examine and reduce heterogeneity (differences in the true treatment effect between trials) and inconsistency (differences in the true treatment effect between direct and indirect evidence) in the network. As a consequence, an IPD network meta-analysis has the potential for more precise, reliable and informative results for clinical practice and even allows treatment comparisons to be made for individual patients and targeted populations conditional on their particular characteristics.

## Introduction

A network meta-analysis (NMA) project compares multiple treatments that have been evaluated in existing randomised trials.^1^ A conventional NMA uses aggregate data (AD) on treatment effects extracted from study publications, such as estimates of odds ratios (ORs), hazard ratios (HRs) or mean differences and corresponding standard errors, or the total participants and events per treatment group. An alternative approach is an NMA of individual participant data (IPD), in which the participant-level data are obtained from multiple studies then checked, harmonised and synthesised.^2^ IPD refers to the raw information recorded for each participant in a study, such as baseline characteristics, prognostic factors, treatments received, outcomes and follow-up details. In this article, we describe the potential benefits that IPD offers for NMA projects compared with using AD, emphasising how IPD enables more precise, reliable and informative results for patients and clinical decision makers. This work adapts and extends our recent book chapter on this topic.^3^

## What is an NMA?

A pairwise meta-analysis compares two treatments (eg, *A* and *B*) by combining the evidence only from trials that directly compared those treatments. However, different trials often evaluate different sets of treatments (eg, some compare *A* and *B*, others compare *A* and *C* or *B* and *C*), and then the pairwise meta-analysis approach is unable to provide a coherent comparison of all treatments. Moreover, for some pairs of treatments there may be no studies comparing them directly at all.

To address this, NMA provides a comprehensive framework to coherently compare and rank all available treatments for a specific disease. It does so by synthesising data from all trials in a single analysis, using both *direct evidence* (eg, about *A vs B* from trials that compared *A* to *B* head-to-head) and *indirect evidence* (eg, about *A vs B* from trials that compared *A* to *C* and trials that compared *B* to *C*).^4–6^ The premise is as follows. For a randomised trial in a particular population of patients, the following relationship holds exactly

Treatment contrast of *A vs B* = (treatment contrast of *A vs C*) – (treatment contrast of *B vs C*)

where ‘treatment contrast’ is the true relative effect between two treatments, measured on a scale such as a log risk ratio, log OR, log HR or mean difference. In an NMA, we assume that this relationship also holds (on average) across the populations of patients in different trials. This notion is often referred to as the consistency assumption (or coherence),^7^ and it allows us to combine direct and indirect evidence from trials comparing different sets of treatments.

The validity of combining studies in a pairwise meta-analysis relies on the assumption that relative treatment effects are exchangeable between trials.^8^ This is appropriate when trials are sufficiently similar with respect to all study-level and patient-level characteristics that might impact on the relative effect of the treatments being compared (eg, quality, length of follow-up, casemix). Exchangeability also underpins the validity of combining direct and indirect evidence in a network meta-analysis (and thus the statistical consistency, ie, the agreement between direct and indirect evidence), and it implies that the relative effects between any pair of treatments observed directly in some trials would be the same in other trials where they are unobserved. This concept is also known as transitivity,^9 10^and when transitivity does not hold, there is likely to be inconsistency (incoherence) in the NMA such that the direct and indirect evidence disagree.

Between-study heterogeneity in treatment effects is another important concept in NMA, which refers to genuine differences in the true treatment effects (eg, for *A vs B*) across trials, and is caused by differences in treatment effect modifiers across trials making the same comparison(s) (eg, trials of *A vs B*). Effect modifiers are methodological or clinical characteristics of trials that influence the magnitude of relative treatment effects on a given scale, and examples include duration of follow-up, outcome definitions, trial quality (risk of bias) and participant-level characteristics.^11–14^

Inconsistency is also a consequence of treatment effect modifiers, but specifically when there are systematic imbalances in effect modifiers across trials making different comparisons. Thus, an effect modifier might differ across the trials in a network (eg, some trials were performed in younger patients, some in older patients, and age impacts on relative treatment effects); this might cause heterogeneity. However, if there are systematic differences in age across comparisons (eg, all *A vs B* studies are in younger patients and all *A vs C* studies are in older patients), this might cause inconsistency.

## Potential benefits of using IPD for NMA

Compared with using AD from existing trials, the availability of IPD brings important advantages for NMA projects, and five key benefits are now described.^3^

### Benefit 1: improving the quality and scope of information available

A key benefit of IPD over AD is the potential to improve the quantity, completeness, and validity of data available for each trial, because there is no need to be limited by the study-level information (ie, AD) that has been published for each trial. This enhances data quality,^15–18^ provides independent scrutiny of the trial data and enables more information to be available for the NMA. This may lead to more reliable and informative conclusions, with potentially reduced heterogeneity and inconsistency.

For instance, there is greater ability to standardise outcome and covariate definitions across trials, and to include outcomes that were not reported in the original publications, or participants who were inappropriately excluded from the original trial analyses.^15–17^ This can help reduce potential trial reporting biases^19^ and increase the quantity of information available for the NMA, boosting the statistical power to compare treatments.^20^ For example, studies of depression often report the outcome ‘response to treatment’, with the definition varying across different studies.^21^ One study might define response as 20% reduction in baseline severity and another study might use a 50% reduction, while a third study might define response as a different endpoint severity being below another arbitrary threshold (on some scale). When using AD we are limited to using these highly variable definitions of response, which may lead to large heterogeneity and greater scope for inconsistency. If IPD are available, we can better harmonise the definition of response and the severity score can be analysed on its continuous scale, thus avoiding dichotomisation and allowing a more powerful investigation of the outcome as originally measured. For example, in their IPD NMA, Karyotaki *et al* used IPD to convert depression scores to the same Patient Health Questionnaire-9 (PHQ-9) scale,^22^ and subsequently chose to analyse PHQ-9 on its continuous scale rather than dichotomising into high-score and low-score groups.

Most IPD NMA projects are collaborative endeavours, involving direct contact with trial investigators, which can help to identify trials not easily identifiable via other forms of searching,^15–17^ and to clarify the eligibility of potentially relevant trials. Trial investigators can also provide extra information leading to more reliable risk of bias assessments than what is achievable from trial reports,^23^ and if potential biases or errors are identified, they may be able to supply additional data to resolve or minimise these.^15–17^ IPD also allow more flexible and detailed modelling of the survival function,^24–26^ potentially including longer follow-up times (or even larger numbers of participants), for example, for those trials that continued monitoring (or recruiting) participants beyond the timepoint when the original analyses were conducted.

### Benefit 2: examining and plotting distributions of covariates across trials

Before undertaking an NMA, it is important to select only those trials relevant to the population of clinical interest and to then identify any systematic differences in variables that might affect measures of relative treatment effect. Of particular interest are suspected effect modifiers or, specifically when analysing ORs or HRs, prognostic factors that modify baseline risk, as these may lead to heterogeneity or inconsistency in the NMA if their distribution is different across trials (see also benefits 4 and 5).

**Figure 1:**
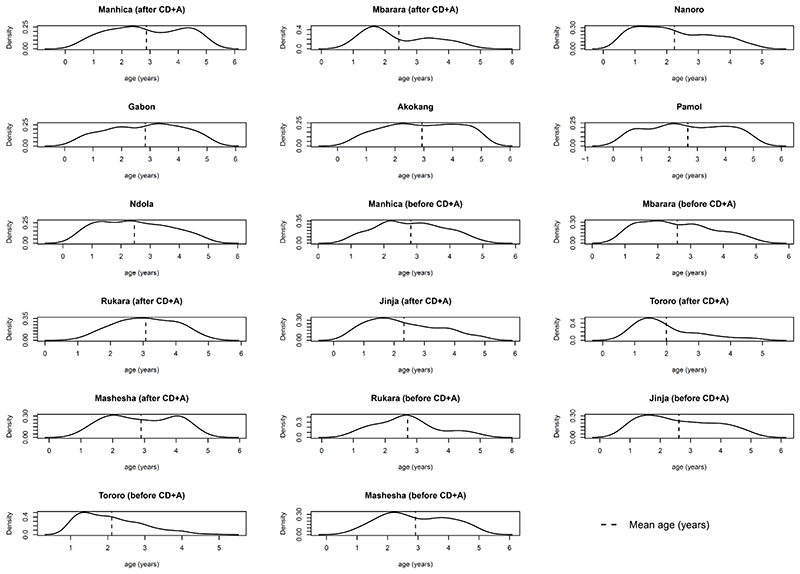
Distribution of age as derived directly from the IPD for each of 17 sites (‘studies’) included in the IPD NMA of Donegan *et al*.^51^ The distribution is similar in most studies, though slightly more skewed in some studies (eg, Mbarara, Tororo), which may lead to heterogeneity or inconsistency in the network due to age. Figure originally presented in online supplemental material of Donegan *et al*.^51^ CD+A, chlorproguanil-dapsone plus artesunate; IPD, individual participant data; NMA, network meta-analysis.

When using only AD, statistical summaries of the distributions of participant-level characteristics need to be extracted from the trial publications (or obtained from the trial investigators). Such AD may be the mean and SD for a continuous covariate, or the proportion of participants in each category for a categorical covariate. In contrast, when IPD are available for each trial, researchers can summarise and plot covariate distributions themselves,^27 28^ and may have access to a broader set of recorded covariates than summarised in the original trial publication. An example is provided in figure 1. This allows better comparison of how covariate distributions differ across comparisons and across trials to help assess their potential to cause heterogeneity and inconsistency in the network.^29^ For example, to assess the plausibility of the consistency assumption in their IPD NMA, Karyotaki *et al* ‘checked the distribution of possible effect size modifiers in the studies grouped by comparison’ and did not identify any systematic differences across comparisons.

### Benefit 3: standardising and improving the analysis of each trial

With IPD, effect measures can be standardised across trials, and this allows NMA researchers to define their own estimands of interest, rather than being limited to those presented in the original trial publications. For example, many published trials report ORs, but with IPD the NMA researcher may rather be able to estimate HRs in order to account for censoring and time-to-event information. Similarly, restricted mean survival time differences could be used rather than HRs if non-proportional hazards are a concern.^30^ Such decisions can be informed by using the IPD to check model assumptions (eg, proportional hazards assumption) and model fit,^31^ after addressing other issues such as outliers, none of which are likely to be possible without IPD.

IPD also enable more appropriate or advanced analytical approaches, in particular for the modelling of time-to-event data^32 33^; the analysis of continuous variables on their continuous scale (rather than dichotomised); the examination of non-linear relationships between covariates and the outcome^34^; the inclusion of prognostic factors and treatment–covariate interactions (see benefits 4 and 5); and the handling of missing data, which is especially important for trials that originally used suboptimal or inappropriate methods to deal with missing values.

IPD also facilitate a multivariate NMA approach, to compare treatments across multiple outcomes simultaneously (eg, benefits and harms; systolic and diastolic blood pressure; surrogate outcomes like disease-free and overall survival; pain scores at 6 and 12 months), while accounting for the (participant-level and study-level) correlation among outcomes.^35^ This can lead to more precise inferences and even change the ranking of treatments,^36 37^ compared with an NMA of each outcome separately. For example, in an NMA of 68 trials comparing 13 active antimanic drugs and placebo for acute mania,^38^ two negatively correlated outcomes of interest were efficacy (defined as the proportion of participants with at least a 50% reduction in manic symptoms from baseline to week 3) and acceptability (defined as the proportion of participants with treatment discontinuation before 3 weeks). When performing a separate NMA for each outcome, carbamazepine ranked as the most effective treatment in terms of response; however, when analysing outcomes jointly in a multivariate NMA, carbamazepine fell to fourth place.

### Benefit 4: adjusting for prognostic factors in the analysis of each trial

IPD allow the NMA to compare treatment effects conditional on (ie, adjusting for) prognostic factors, which is important.^2^ First, adjustment for prognostic factors in a single trial (eg, using regression) is often preferred to increase power to detect treatment effects (and, for continuous outcomes, to increase the precision of the estimates), as prognostic factors may explain variation in outcomes across participants,^39–42^ and is necessary to obtain correct estimates of uncertainty when stratified randomisation has been employed^43^; thus, subsequent NMA results may also be more powerful and appropriately quantify uncertainty.^44^ Second, conditional treatment effects also align more closely with the drive toward personalised medicine tailoring treatment decisions to each patient given their particular characteristics and outcome risks. Third, conditioning on observed prognostic factors improves the plausibility of the missing at random assumption for participants with missing outcomes, and so improves on a complete-case analysis of unadjusted treatment effect estimates.

Fourth, adjustment for prognostic factors can improve homogeneity and consistency of treatment effects in an NMA. Even in situations where there are no effect modifiers, differences in the distribution of prognostic factors between trials can lead to heterogeneity and inconsistency to when the treatment effect is measured on the OR or HR scale.^3^ IPD help to address this by adjusting for prognostic factors in the analysis of each trial, and an example is provided in box 1. Occasionally a prognostic factor may also be an effect modifier,^45^ which can also be better modelled using IPD, as considered in benefit 5.

### Benefit 5: including treatment–covariate interactions (effect modifiers)

One of the most important advantages of IPD is that it allows the examination and inclusion of participant-level effect modifiers (treatment–covariate interactions) that would otherwise cause heterogeneity or inconsistency in the NMA. Single trials are rarely powered to detect treatment–covariate interactions, and typically an AD meta-analysis can only examine across-trial relationships, which are prone to aggregation bias and trial-level confounding.^46 47^ In contrast, IPD meta-analysis allows participant-level relationships to be modelled directly and more precisely, for example in a regression that models a covariate's interaction with treatment effect while adjusting for the covariate's prognostic effect (and potentially also other prognostic factors). This allows NMA results to compare treatments for specific patient populations, subgroups or at covariate values defined by participant-level characteristics. Indeed, since the magnitude of treatment effects may change in the presence of effect modification, the best ranking treatment(s) may differ across populations, subgroups, and covariate values defined by effect modifiers.^3 13 48–50^ In this situation, treatment recommendations will need to be tailored to a chosen target population, or for subgroups or individual patients, according to their corresponding (distribution of) covariate values. This type of analysis (adjustment for effect modifiers, followed by the production of population-targeted treatment effect estimates) is known as ‘population adjustment’.^49^

Riley *et al* describe how to include participant-level treatment–covariate interactions in two-stage or one-stage IPD meta-analysis models with a single pairwise comparison.^2 47^ These can be extended to NMA situations to accommodate treatment–covariate interactions corresponding to the multiple treatment effects. Ideally, these interactions are assumed to be independent (ie, different for each treatment), but to aid model convergence, it may be necessary to assume interactions are exchangeable (eg, by including random effects) or even common for each treatment.^51^

**Figure 2:**
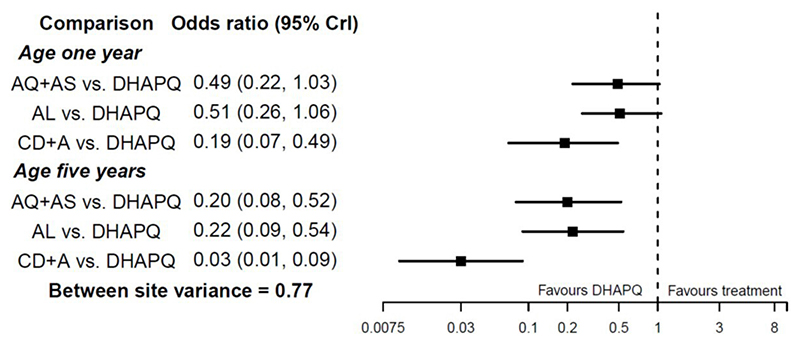
Results reported by Donegan *et al*^51^ after estimation of an NMA allowing for treatment–age interactions, as applied to compare four treatments for malaria using IPD from 11 sites. Figure adapted from Donegan *et al*,^51^ and results assume treatment–age interactions are independent for each treatment comparison. AQ+AS, amodiaquine-artesunat; AL, artemether-lumefantrine; DHAPQ, dihydroartemisinin-piperaquine; IPD, individual participant data; NMA, network meta-analysis.

Consider an NMA presented by Donegan *et al*,^51^ who use IPD to examine four artemisinin-based combination therapies for uncomplicated malaria: amodiaquine-artesunate, dihydroarte-misinin-piperaquine (DHAPQ), artemether-lumefantrine (AL), and chlorproguanil-dapsone plus artesunate. The binary outcome of interest was treatment success at 28 days. IPD were available from 17 sites, which for simplicity can be considered as 17 ‘trials’ here. Age was prespecified as a potential treatment effect modifier, since in areas with endemic malaria older patients are more likely to achieve success on treatment because they have greater immunity. There is strong evidence of an interaction between treatment effects and age. This leads to larger summary treatment effects at higher ages (figure 2). For example, the summary OR for AL versus DHAPQ is 0.51 (95% CI 0.26 to 1.06) at 1year of age, and 0.22 (95% CI 0.09 to 0.54) at 5years of age, when assuming interactions are independent for each treatment comparison.

**Figure 3:**
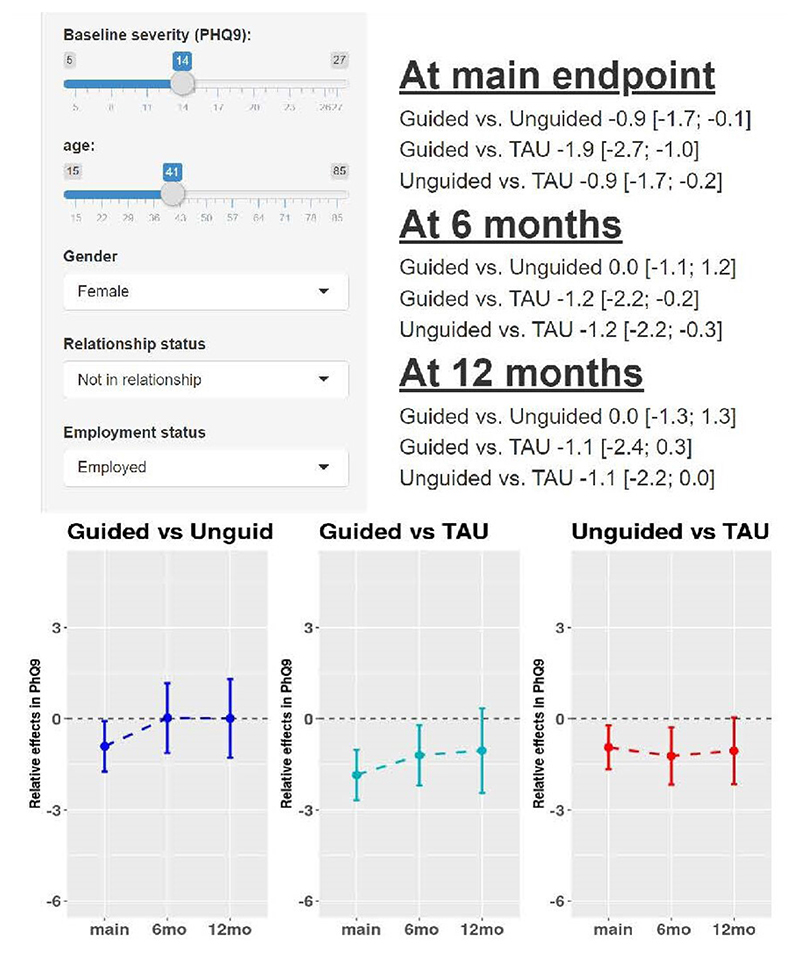
Example output from an online calculator of treatment effects for depression (measured on Patient Health Questionaire-9 (PHQ-9) scale) conditional on patient-level characteristics, as derived from the IPD NMA results of Karyotaki *et al*.^22^ The tool is available here: https://esm.ispm.unibe.ch/shinies/iCBT/). iCBT, internet-based cognitive behavioural therapy; IPD, individual participant data; NMA, network meta-analysis; TAU, treatment as usual.

Karyotaki *et al*^22^ provide an online calculator for estimating and comparing treatment effects conditional on participant-level characteristics of baseline PHQ-9 score, age, gender, relationship status and employment status following their IPD NMA. The tool is illustrated in figure 3. Such predictions often require shrinkage and penalisation techniques,^47 52 53^ in order to mitigate against overfitting (extreme predictions), and Karyotaki *et al* used the least absolute shrinkage and selection operator (LASSO) for this purpose.^22^

## Potential challenges

IPD meta-analysis projects also face challenges.^2^ They are a considerable undertaking, often taking upwards of 2years to obtain, check, harmonise and synthesise IPD. Negotiating and maintaining collaboration with trial investigators takes considerable effort, and care is needed to arrange and adhere to data-sharing agreements, including how IPD are transferred and stored. To safeguard against future conflicts, data-sharing agreements should make clear that the central IPD NMA research team are responsible for making final decisions (eg, about design, IPD included, risk of bias judgements, analysis methods), while still valuing advice from the trial investigators. An independent advisory group may facilitate this.

Inevitably, the requested IPD may not be available from all studies, leading to availability bias concerns,^54^ and the need for methods to combine IPD and AD.^48 50 55^ Multilevel network metaregression extends the IPD NMA framework to incorporate IPD and AD (with full-IPD NMA as a special case),^48^ and is implemented in the multinma R package.^56^ The approach avoids aggregation bias by integrating the individual-level regression model over the covariate distributions in each aggregate study population, and can produce estimates in any target population of interest. Other methods including matching-adjusted indirect comparison, simulated treatment comparison and predictive-adjusted indirect comparison have also been proposed for ‘population adjustment’ with limited IPD, but are limited to a two-study indirect comparison (one IPD and one AD study) and can only produce estimates relevant to the population of the AD study.^49^

## Concluding remarks

In summary, the use of IPD adds value to NMA projects by improving quality (eg, through improved homogeneity and consistency in the network) and scope (eg, additional outcomes and longer follow-up), leading to more reliable and tailored NMA results for clinical practice. In the coming years, we anticipate further methodological research to improve and extend IPD NMA projects, and the website http://www.ipdma.co.uk provides signposts to new methodological developments.

## Data Availability

Data sharing not applicable as no datasets generated and/or analysed for this study. The work presented simply discusses and shares examples from published work, and therefore, no actual individual-level data are available for sharing.

## Box 1 Example of how heterogeneity and inconsistency can arise in an NMA when the distribution of prognostic factors differs across trials

Consider a hypothetical example where, regardless of the trial, the underlying (true) probability (*P*) of having an adverse outcome depends on whether treatment *B* or *A* is used and, in particular, whether a participant smokes, as defined by the equation,

logit (*p*) = –0.5 + (4 × *smoker*) – (0.5 × *B*)

where *B* = 1 if in treatment group *B* and *B* = 0 if treatment group *A*, and *smoker* = 1 if a participant currently smokes and 0 otherwise. Hence, after adjusting for the strong prognostic effect of smoking, the true treatment effect for *B vs A* is an OR of exp(−0.5)=0.61. Note that there are no treatment effect modifiers (eg, no treatment-smoker interaction).

Now consider that two randomised trials of *B vs A* were conducted: trial 1 used a population where 20% of participants smoked, and trial 2 had a population where 80% of participants smoked. Regardless of the proportion of smokers, the equation tells us that both trials have a true OR of 0.61 for the treatment effect adjusted for the prognostic effect of smoking.

However, when ignoring smoking, the unadjusted treatment effect is considerably different in the two trials. The true unadjusted OR for *B vs A* is 0.69 for trial 1 and 0.77 for trial 2. Hence, there is genuine between-trial heterogeneity in the unadjusted OR for trials 1 and 2 (as available in a typical AD NMA), but no heterogeneity in the OR adjusted for smoking (as available from an IPD NMA).

Similarly, if the distribution of smokers is different in each subset of trials that provide direct and indirect evidence for a particular comparison, then the unadjusted ORs will differ for each subset, leading to inconsistency in the NMA for that comparison. However, with IPD such inconsistency can be removed by synthesising ORs adjusted for smoking for each trial.

AD, aggregate data; IPD, individual participant data; NMA, network meta-analysis.

## What Is Already Known On This Topic

⇒ A network meta-analysis provides a framework to compare and rank all available treatments for a specific disease, using both direct evidence and indirect evidence. Most network meta-analysis projects use aggregate data obtained from study publications or study authors.

## What This Study Adds

⇒ The use of individual participant data (IPD), rather than aggregate data, can enhance network meta-analysis projects by increasing the quality and scope of information available; by allowing participant characteristics to be compared across studies; by standardising and improving statistical analyses; and by inclusion of prognostic factors and treatment effect modifiers to reduce concerns of inconsistency and heterogeneity.

## How This Study Might Affect Research, Practice Or Policy

⇒ The use of IPD in network meta-analysis projects can produce more precise, reliable and informative results for patients and clinical decision makers. We recommend network meta-analysis projects consider using IPD where possible.
